# Secretoglobin expression in ovarian carcinoma: lipophilin B gene upregulation as an independent marker of better prognosis

**DOI:** 10.1186/1479-5876-11-162

**Published:** 2013-07-02

**Authors:** Eliana Bignotti, Renata A Tassi, Stefano Calza, Antonella Ravaggi, Elisa Rossi, Carla Donzelli, Paola Todeschini, Chiara Romani, Elisabetta Bandiera, Laura Zanotti, Mario Carnazza, Francesco Quadraro, Germana Tognon, Enrico Sartori, Sergio Pecorelli, Dana M Roque, Alessandro D Santin

**Affiliations:** 1 Angelo Nocivelli Institute of Molecular Medicine, Division of Gynecologic Oncology, University of Brescia, Viale Europa 11, 25123 Brescia, Italy; 2 Department of Molecular and Translational Medicine, University of Brescia, Brescia, Italy; 3 Department of Medical Epidemiology and Biostatistics, Karolinska Institutet, Stockholm, Sweden; 4 Centro de Investigaciones Biologicas, Consejo Superior de Investigaciones Cientificas (CSIC) and Centro de Investigación Biomédica en Red de Enfermedades Raras (CIBERER), Madrid, Spain; 5 Department of Pathology, University of Brescia, Brescia, Italy; 6 Department of Obstetrics, Gynecology & Reproductive Sciences, Yale University School of Medicine, New Haven, CT 06520-8063, USA

**Keywords:** Ovarian carcinoma, Secretoglobins, Lipophilin B, Gene expression, Prognosis, Biomarker

## Abstract

**Background:**

The aim of the present study was to investigate within ovarian carcinoma and normal ovarian biopsies the gene expression of multiple secretoglobin family members relative to mammaglobin B, which we previously reported as a promising novel ovarian carcinoma prognostic marker.

**Methods:**

Using quantitative real-time Reverse Transcription PCR we tested 53 ovarian carcinoma and 30 normal ovaries for the expression of 8 genes belonging to the secretoglobin family: mammaglobin A, lipophilin A, lipophilin B, uteroglobin, HIN-1, UGRP-1, RYD5 and IIS. Next, we decided to expand the LipB gene expression analysis to a further 48 ovarian carcinoma samples, for a total of 101 tumor tissues of various histologies and to study its protein expression by immunohistochemistry in formalin-fixed paraffin-embedded tumors and normal ovaries. Finally, we correlated lipophilin B gene and protein expression to conventional patient clinico-pathological features and outcome.

**Results:**

We found significant mammaglobin A, lipophilin A, lipophilin B and RYD5 gene overexpression in ovarian carcinomas compared to normal ovaries. Lipophilin B mRNA showed a higher presence in tumors (75.4%) compared to normal ovaries (16.6%) and the most significant correlation with mammaglobin B mRNA (r_s_ =0.77, p < 0.001). By immunohistochemical analysis, we showed higher lipophilin B expression in the cytoplasm of tumor cells compared to normal ovaries (p < 0.001). Moreover, lipophilin B gene overexpression was significantly associated with serous histology (serous vs clear cell p = 0.027; serous vs undifferentiated p = 0.007) and lower tumor grade (p = 0.02). Lower LipB mRNA levels (low versus high tertiles) were associated to a shorter progression-free (p = 0.03, HR = 2.2) and disease-free survival (p = 0.02, HR = 2.5) by univariate survival analysis and, importantly, they remain an independent prognostic marker for decreased disease-free (p = 0.001, HR = 3.9) and progression-free survival (p = 0.004, HR = 2.8) in multivariate Cox regression analysis.

**Conclusions:**

The present study represents the first quantitative evaluation of secretoglobin gene expression in normal and neoplastic ovarian tissues. Our results demonstrate lipophilin B gene and protein upregulation in ovarian carcinoma compared to normal ovary. Moreover, lipophilin B gene overexpression correlates with a less aggressive tumor phenotype and represents a novel ovarian carcinoma prognostic factor.

## Background

Epithelial ovarian cancer (EOC) represents the fifth leading cause of cancer deaths among women, with 65,538 estimated new cases and 42,704 deaths in Europe during the 2012 [1]. The high mortality rate of EOC reflects its asymptomatic nature, a lack of adequate screening tests, the frequent diagnosis at late stage, and the frequency of chemoresistant recurrences [2]. Unfortunately, EOC is fundamentally an incurable disease, and currently available prognostic parameters are not able to adequately predict its relapse and clinical course [3]. There is a critical need to discover outcome-informative biomarkers to select those EOC patients who might benefit from individualized targeted therapies.

Secretoglobins are secreted proteins of small molecular weight belonging to a polypeptide family that includes at least nine family members in humans: SCGB1A1 (Uteroglobin or Clara cell 10 kDa protein [CC10]), SCGB1C1 (ligand binding protein RYD5 [RYD5]), SCGB1D1 (lipophilin A [LipA]), SCGB1D2 (lipophilin B [LipB] or BU101), SCGB1D4 (interferon-γ inducible SCGB [IIS]), SCGB2A1 (mammaglobin 2 [MGB2] or lipophilin C), SCGB2A2 (mammaglobin 1 [MGB1] or uteroglobin 2 [UGB2]), SCGB3A1 (uteroglobin-related protein 2 [UGRP2] or high-in-normal [HIN1]) and SCGB3A2 (uteroglobin-related protein 1 [UGRP1]) [4-7]. Although the first secretoglobin polypeptide was discovered more than 30 years ago, the pathophysiological functions of the family are still poorly known. Secretoglobin expression has generally been observed in secretory epithelia [8-11] and their dysregulated expression has been reported in several malignancies, such as lung [12-14], pituitary [15], breast [16,17] and gynecological cancers [18-20].

Our group has recently reported mammaglobin B (MGB2) as highly expressed both at the gene and protein level in EOC tissues [21,22]. Moreover, in a subsequent study we demonstrated that MGB2 expression characterizes a less aggressive EOC phenotype and is correlated with reduced risk of recurrence, suggesting its promising role as a novel marker for EOC prognosis [23]. To the best of our knowledge, no studies have extensively investigated the gene expression of secretoglobins in EOC tissues and normal ovarian controls. Because of this lack of information, in the present study we quantified for the first time gene expression of all prominent human secretoglobin family members in a panel of fresh-frozen EOC and normal ovarian biopsies. Since lipophilin B (LipB) mRNA showed the highest correlation with MGB2, we further validated LipB gene expression in an expanded set of samples and, subsequently, LipB protein expression by immunohistochemistry. Finally, we correlated LipB gene and protein expression to conventional clinicopathological features and outcome endpoints in a selected group of homogeneously-treated EOC patients to determine its prognostic significance in EOC.

## Methods

### Patients

Cancer tissue samples were obtained from 101 patients diagnosed with EOC and treated at the Division of Gynecologic Oncology of the University of Brescia (Italy), between June 2003 and December 2010. The study has been performed following the Declaration of Helsinki set of principles and it has been approved by the Research Review Board- the Ethic Committee- of the Spedali Civili, Brescia, Italy (study reference number: 527/B4/4).

Each patient underwent primary cytoreductive surgery including total abdominal hysterectomy, bilateral salpingo-oophorectomy, omentectomy and pelvic and periaortic lymph node sampling, with cytological evaluation of ascites or peritoneal washings. Age, histological type, stage, grade, residual tumor, presence of ascites and lymph node involvement were recorded in all cases. Tumor staging was in accordance with the International Federation of Gynecology and Obstetrics (FIGO) criteria, while tumor grade and histological type were determined following World Health Organization (WHO) standards; optimal cytoreduction was defined as no macroscopic residual tumor after primary surgery (RT = 0).

Ninety-two EOC patients received the same postoperative platinum-based chemotherapy and were included in survival analysis. Specifically, 80% of patients received carboplatin and paclitaxel-combined regimen, 15% were treated with carboplatin only and the remaining 5% of patients had a platinum-based regimen combined with other drugs (i.e. topotecan, gemcitabine). Patients were followed from the date of surgery until death or the last observation (median follow-up 39 months, range = 7–93 months). At the time of last follow-up, 31 patients (34%) were alive without evidence of disease, 15 patients (16%) were alive with disease and 46 patients (50%) were dead of disease.

### Tissue collection and establishment of HOSE cell lines

All EOC specimens were collected and immediately frozen as previously described [21]. Only samples containing at least 70% of tumor epithelial cells as assessed by a staff pathologist were used for total RNA extraction.

Control specimens included 10 normal human ovarian surface epithelial short-term cultures (HOSE), 10 fresh-frozen normal ovarian biopsies and 10 normal ovarian surface epithelium brushings. HOSE were cultured from normal ovaries of patients undergoing hysterectomy and bilateral salpingo-oophorectomy for benign pathologies, as previously reported [21] and only cell cultures containing at least 99% epithelial cells were retained for total RNA extraction. Normal ovaries were manually sharply macrodissected and immediately frozen in liquid nitrogen. Ovarian brushings were performed on the whole normal ovary with a cell culture scraper in phosphate buffered saline (PBS) and cells were collected by centrifugation at 1000 rpm for 10 minutes. The cell pellet was then resuspended with 200 μl TRIzol Reagent (Invitrogen life Technologies, Carlsbad, CA, USA) and immediately frozen at −20°C for further RNA extraction.

### Total RNA extraction and quantitative real-time reverse transcription polymerase chain reaction (qRT-PCR)

Total RNA was extracted and purified from 101 EOC tissues with different histologies, 10 HOSE, 10 fresh-frozen normal ovarian biopsies and 10 normal ovarian surface brushings. Total RNA extraction and quality control were performed as previously reported [21]. Since HIN-1 assay (Hs00369360_g1) is designed with sequences that are complementary also to genomic DNA, RNA samples were treated with TURBO DNase enzyme (TURBO DNA-free Kit; Ambion, Inc. Applied Biosystem Business, CA) to remove contaminating DNA eventually present. In particular, four μg of total RNA were digested with 2U of TURBO DNase enzyme in a 25 μl-reaction for 30 minutes at 37°C. The digestion was stopped by adding 2.5 μl of DNase Inactivation Reagent, followed by centrifugation. One μg of extracted RNA was reverse-transcribed using random hexamers in a final volume of 20 μl according to the SuperScript TM II RT RnaseH-reverse transcriptase protocol (Invitrogen life Technologies, Carlsbad, CA, USA).

QRT-PCR was performed on the ABI PRISM 7000 Sequence detection System (Applied Biosystem, Applera UK, Cheshire, UK) using the TaqMan Universal PCR master mix and the following Assay on Demand primers and probes (Applied Biosystem): Hs00255208_m1 (lipophilin B), Hs00267190_m1 (mammaglobin A), Hs00255210_m1 (lipophilin A), Hs00171092_m1 (uteroglobin), Hs00369360_g1 (HIN-1), Hs00369678_m1 (UGRP-1), Hs00377337_m1 (SCGB1C1) and Hs00419570_m1 (SCGB1D4). Reaction and thermal cycling conditions were performed as previously reported [21]. The comparative threshold cycle (Ct) method was used for the calculation of amplification fold and the delta-delta Ct method was used to obtain relative gene expression values, normalized using glyceraldehyde-3-phosphate dehydrogenase (GAPDH) as an internal control.

### Immunohistochemistry on formalin-fixed tissues

Immunohistochemistry (IHC) was performed on 100 formalin fixed-paraffin embedded EOC tissues of different histologies and 10 normal ovaries. Both tumor and normal samples were matched with the ones analyzed at the mRNA level by qRT-PCR. Antigen retrieval was performed in EDTA buffer pH 8 by using microwave at 750 W. Anti-lipophilin B antibody diluted 1:10 (S-17 goat polyclonal antibody, Santa Cruz Biotechnology, Inc, Santa Cruz, CA, USA) was applied for 45 minutes, followed by a secondary biotinylated anti-goat antibody diluted 1:20 (Vector Laboratories, Burlingame, CA) and the streptavidin-biotin complex (StreptABComplex/HRP, Dako, CA, USA). 3’3-diaminobenzidine (Dako, CA, USA) was used as chromogen and the sections were counterstained by hematoxylin (Dako).

Slides were analyzed at medium/high power view (20x and 40x magnification) and a scoring method based either on the intensity of the staining or on the percentage of positive tumor cells was applied as follows: intensity was scored 0 (negative), 1 (weak), 2 (moderate), 3 (strong), while percentage of positive cells was scored as 0 (0%), 1 (1-10%), 2 (11-50%), 3 (51-100%). A single scale with scores 0–9 was obtained multiplying the intensity and the percentage staining score and a total score was calculated grouping score 0 in total score 0, 1–3 in total score 1, 4 and 6 in total score 2 and 9 in total score 3.

### Statistical analysis

The variation in secretoglobin gene expression measured by qRT-PCR between EOCs and healthy controls and among EOC histotypes was evaluated using Wilcoxon-Mann–Whitney test and a standard parametric ANOVA, respectively. The difference in LipB immunohistochemical staining between groups, considered on the ordinal scale, was investigated using Wilcoxon-Mann–Whitney test and with a nonparametric one-way ANOVA. Approximate p-values were computed through Monte Carlo resampling (using B = 9999 replications). Multiple comparison correction was applied for paired tests using a step-down procedure. The correlation between LipB expression measured by qRT-PCR and IHC staining was evaluated by means of the polyserial correlation coefficient. The association between LipB mRNA expression and clinicopathologic parameters was investigated with an ANOVA (after log transformation). The association between IHC, coded as ≤1, 2 and 3 and clinical covariates were evaluated by means of Kruskal-Wallis and Wilcoxon-Mann-Whithney tests. For survival analysis, three endpoints (cancer relapse, cancer progression and death due to cancer) were used to calculate disease-free survival (DFS), progression-free survival (PFS) and disease-specific overall survival (OS), respectively. DFS was defined as the time interval between the date of surgery and the date of identification of disease recurrence, PFS was defined as the time interval between the date of surgery and the date of identification of progressive disease (disease not treatable with curative intent) and OS was defined as the time interval between the date of surgery and the date of death. For all three endpoints the last date of follow-up was used for censored subjects. Survival models were fitted using the Cox proportional hazard model, while survival curves were drawn based on the Kaplan-Meier methods. The impact of LipB qRT-PCR expression on prognosis was evaluated categorizing the qRT-PCR values in tertiles computed on the whole cohort (Low: <891; Medium: 891–1782.9;? High: > 1782.9) while immunostaining was analyzed both as a continuous variable and by group (≤1, 2 and 3). Optimal threshold in ROC curve was computed as the marker value that minimizes the distance from the top left corner (sensitivity & specificity equal to 100%) and the curve. In all analyses, a *p* value <0.05 was considered significant. All statistical analyses were performed using the R language.

## Results

### Evaluation of secretoglobin gene expression by qRT-PCR

Secretoglobin gene expression was evaluated on 53 EOC tissues and 30 normal controls (NO), including 10 HOSE, 10 fresh-frozen normal ovarian biopsies and 10 normal ovarian surface epithelium brushings. As displayed in Table 1, there was no difference in gene expression between EOC and NO for uteroglobin, HIN-1, UGRP-1 and IIS. Conversely, RYD5, mammaglobin A, lipophilin A, and lipophilin B genes were overexpressed in EOCs compared to NO. The optimal cut-off point of positivity in gene expression for each secretoglobin was determined by means of a receiver operating characteristic curve, and it was set at relative quantification = 0.5 for uteroglobin, UGRP-1, IIS, RYD5, mammaglobin A and lipophilin A, at relative quantification = 2.5 for HIN-1, at relative quantification = 2559.5 for mammaglobin B and, finally, at relative quantification = 2520.5 for Lipophilin B.

**Table 1:** Distribution of secretoglobin mRNA expression in normal ovaries (NO) and epithelial ovarian cancers (EOC) and correlation with MGB2 gene expression^*^

**Secretoglobin**	**% expressing**	**Relative mRNA expression **	**p value**	**Correlation with SCGB2A1 gene expression**	
		**Mean (SD)**	
				**Rho cc**	**p value**
SCGB2A1 (Mammaglobin B)	EOC 46/53 = 86.7%	186883.54 (63045.00)	**p < 0.001**	1	-
	NO 2/23 = 8.6%	1432.00 (648.59)			
SCGB2A2 (Mammaglobin A)	EOC 36/53 = 67.9%	23295.42 (21171.01)	**p < 0.001**	0.63	**p < 0.001**
	NO 2/30 = 6.6%	0.37 (0.33)			
SCGB1D1 (Lipophilin A)	EOC 40/53 = 75.4%	4741.58 (2680.96)	**p < 0.001**	0.66	**p < 0.001**
	NO 2/30 = 6.6%	5.83 (4.19)			
SCGB1D2 (Lipophilin B)	EOC 40/53 = 75.4%	122631.77 (48990.82)	**p < 0.001**	0.77	**p < 0.001**
	NO 5/30 = 16.6%	3818.80 (1908.12)			
SCGB3A1 (HIN-1)	EOC 8/53 = 15.1%	386,48 (1606,95)	p = 0.21	0.39	**p < 0.001**
	NO 3/30 = 10.0%	2194.48 (6305.41)			
SCGB1C1 (RYD5)	EOC 37/53 = 69.8%	57.79(26.83)	**p < 0.001**	0.36	**p < 0.001**
	NO 3/30 = 10%	2.70 (2.14)			
SCGB1D4 (IIS)	EOC 29/53 = 54.7%	72084.23 (69346.5)	p = 0.411	0.50	**p < 0.001**
	NO 10/26 = 38.4%	340.77 (158.75)			
SCGB1A1 (Uteroglobin)	EOC 11/53 = 20.7%	138.72 (117.09)	p = 0.452	0.25	**p = 0.03**
	NO 5/30 = 16.6%	185.43 (116.76)			
SCGB3A2 (UGRP-1)	EOC 6/53 = 11.3%	2.85 (1.65)	p = 0.190	0.16	p = 0.17
	NO 1/30 = 3.3%	0.17 (0.16)			

^*^ The p value indicates significance in the difference between NO and EOC for each secretoglobin mRNA expression (ns = not significant, SD = standard deviation, Rho cc = Spearman Rho correlation coefficient).

**Figure 1:**
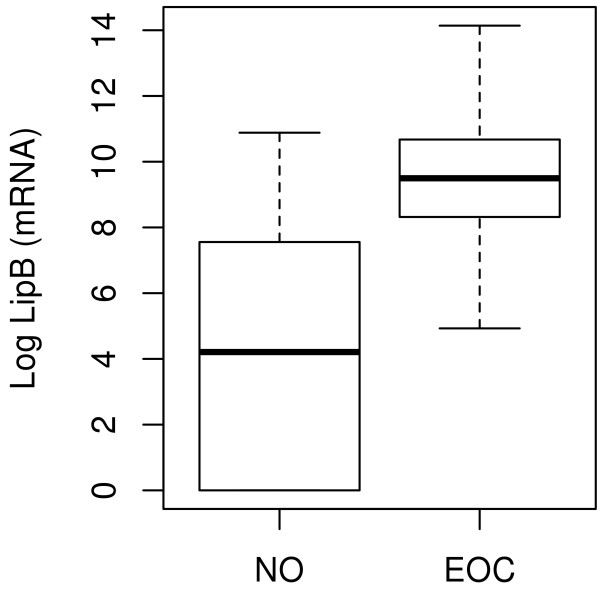
**Lipophilin B mRNA relative expression in epithelial ovarian cancers (EOC) compared to normal ovaries (NO).** LipB qRT-PCR analysis in a total of 101 EOCs of various histologies and in 30 NOs confirmed its overexpression in tumor tissues compared to healthy controls (fold change = 84.8; p < 0.001).

LipB mRNA showed the most significant correlation with mammaglobin B mRNA (r_s_ =0.77, p < 0.001), the expression of which was previously published by our group on the same cohort of patients [23] (reported in Table 1 in the present study). LipB mRNA was highly expressed in EOC (75.4%) compared to normal controls (16.6%). Consequently, we decided to expand the LipB qRT-PCR analysis to further 48 EOC samples, for a total of 101 tumor tissues of various histologies, and its overexpression was confirmed in ovarian tumor tissues compared to healthy controls (fold change = 84.8; p < 0.001), as displayed in Figure 1. According to the chosen threshold, 5 out of 30 NO and 80 out of 101 tumor samples were positive for LipB mRNA expression. The sensitivity and specificity of the test were therefore 80.8% and 83.3%, respectively.

### Validation of LipB protein expression by immunohistochemical staining

**Figure 2:**
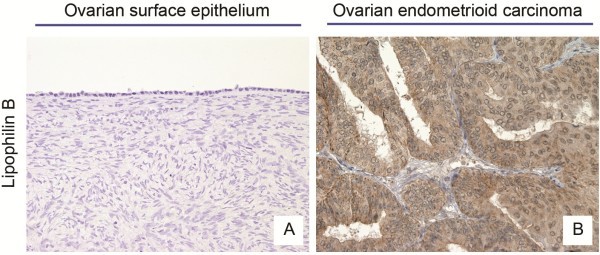
**Lipophilin B immunohistochemical staining in epithelial ovarian cancers (EOC) compared to normal ovaries (NO).** Immunohistochemistry showed no expression in normal ovaries **(A)**, while it displayed a predominant staining in the cytoplasm of ovarian carcinoma cells **(B)**.

To confirm LipB gene expression at the protein level, immunohistochemistry was carried out on 100 primary EOCs of different histological types and 10 normal ovaries. Cytoplasmatic staining for LipB was detected in 95 out of 100 (95%) ovarian cancer specimens (Figure 2B), with the majority of primary EOCs (59%) showing a strong to moderate staining (score 2 and 3, Table 2). On the contrary, none of the 10 normal ovaries showed any immunoreactivity for LipB, neither in the surface epithelium nor in the stroma (Figure 2A). In all EOCs, LipB immunoreactivity localized exclusively to the cytoplasm of neoplastic epithelium; stromal cells were negative. No significant difference in LipB protein expression among different EOC histotypes was found. Finally, the correlation between LipB mRNA expression and IHC staining performed in paired tumor samples was not significant (p = 0.811).

**Table 2:** Clinico-pathologic characteristics of 100 EOC patients and their association to LipB protein expression

**LipB protein expression**					
	**Total**	**score ≤ 1**	**score = 2**	**score = 3**	**p-value**
		**n(%)**	**n(%)**	**n(%)**	
**Age at diagnosis (y)**					
≤60	48	22 (46)	20 (42)	6 (12)	0.6
>60	52	19 (37)	25 (48)	8 (15)	
**Histological type**					
serous	51	25 (49)	17 (33)	9 (18)	
endometrioid	17	5 (29)	11 (65)	1 (6)	
clear cell	11	6 (55)	3 (27)	2 (18)	
mucinous	3	0	1 (33)	2 (67)	0.3
mixed	13	4 (31)	9 (69)	0	
undifferentiated	5	1 (20)	4 (80)	0	
**FIGO stage**					
I + II	27	10 (37)	13 (48)	4 (15)	
III + IV	73	31 (42)	32 (44)	10 (14)	0.7
**Histological grade**					
G1	5	1 (20)	2 (40)	2 (40)	
G2 + G3	95	40 (42)	43 (45)	12 (13)	0.2
**Residual tumor (RT), cm**					
RT = 0	40	14 (35)	21 (52)	5 (13)	
RT > 0	60	27 (45)	24 (40)	9 (15)	0.5
**Presence of ascites**					
no	44	19 (43)	18 (41)	7 (16)	
yes	56	22 (39)	27 (48)	7 (13)	0.5
**Lymph nodal involvement**					
negative	51	16 (31)	23 (45)	12 (24)	
positive	26	13 (50)	12 (46)	1 (4)	0.3
missing	23	12 (52)	10 (44)	1 (4)	

### LipB expression and clinicopathologic variables

**Figure 3:**
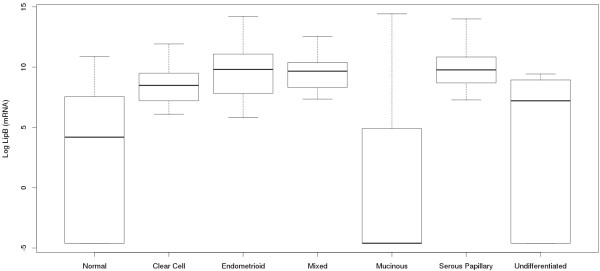
**Lipophilin B mRNA relative expression in EOC different histotypes.** LipB expression was significantly higher in serous and mixed tumors compared to undifferentiated histotype (p = 0.007 and p = 0.036, respectively) and in serous tumors compared to clear cell histotype (p = 0.027).

No significant correlation was found between LipB protein expression and clinicopathological variables (Table 2), however, LipB gene overexpression was significantly associated with lower tumor grade (p = 0.02) and histological type (Table 3). In particular, LipB gene expression was significantly higher in serous EOC compared to clear cell histotype and in serous and mixed tumors compared to undifferentiated histotype (Figure 3, p = 0.027, p = 0.007 and p = 0.036, respectively).

**Table 3:** Clinico-pathologic characteristics of 101 EOC patients and their association with LipB mRNA expression

**Parameters**	**Fold change**	**95%CI**	**p-value**
**Histological type**			
Serous vs endometrioid	0.84	0.25-2.22	0.773
Serous vs clear cell	3.43	0.002-0.79	**0.027**
Serous vs mucinous	1756	0.66-3.83	0.345
Serous vs mixed	1.05	0.51-2.34	0.533
Serous vs undifferentiated	12.99	1.80-7.88	**0.007**
Endometrioid vs clear cell	4.04	0.66-2.83	0.265
Endometrioid vs mucinous	2069	0.46-3.73	0.487
Endometrioid vs mixed	1.24	0.27-2.32	0.765
Endometrioid vs undifferentiated	15.31	0.96-1.73	0.094
Clear cell vs mucinous	511	0.36-3.52	0.446
Clear cell vs undifferentiated	3.78	0.46-2.94	0.269
Mixed vs clear cell	3.25	0.71-3.72	0.151
Mixed vs mucinous	1660	0.36-3.54	0.412
Mixed vs undifferentiated	12.28	0.007-0.89	**0.036**
Undifferentiated vs mucinous	135	0.22-3.77	0.990
**Histological grade**			
G2 + G3 vs G1	0.04	0.003-0.59	**0.02**
**FIGO stage**			
III + IV vs I + II	0.80	0.29-2.14	0.66
**Age at diagnosis (y)**			
>60 vs ≤60	1.09	0.512-2.335	0.58
**Residual tumor (RT), cm**			
RT > 0 vs RT = 0	0.99	0.41-2.38	0.99
**Presence of ascites**			
yes vs no	1.69	0.76-3.73	0.19
**Lymph node involvement**			
positive vs negative	1.03	0.42-2.52	0.94

### LipB expression and patient survival

**Figure 4:**
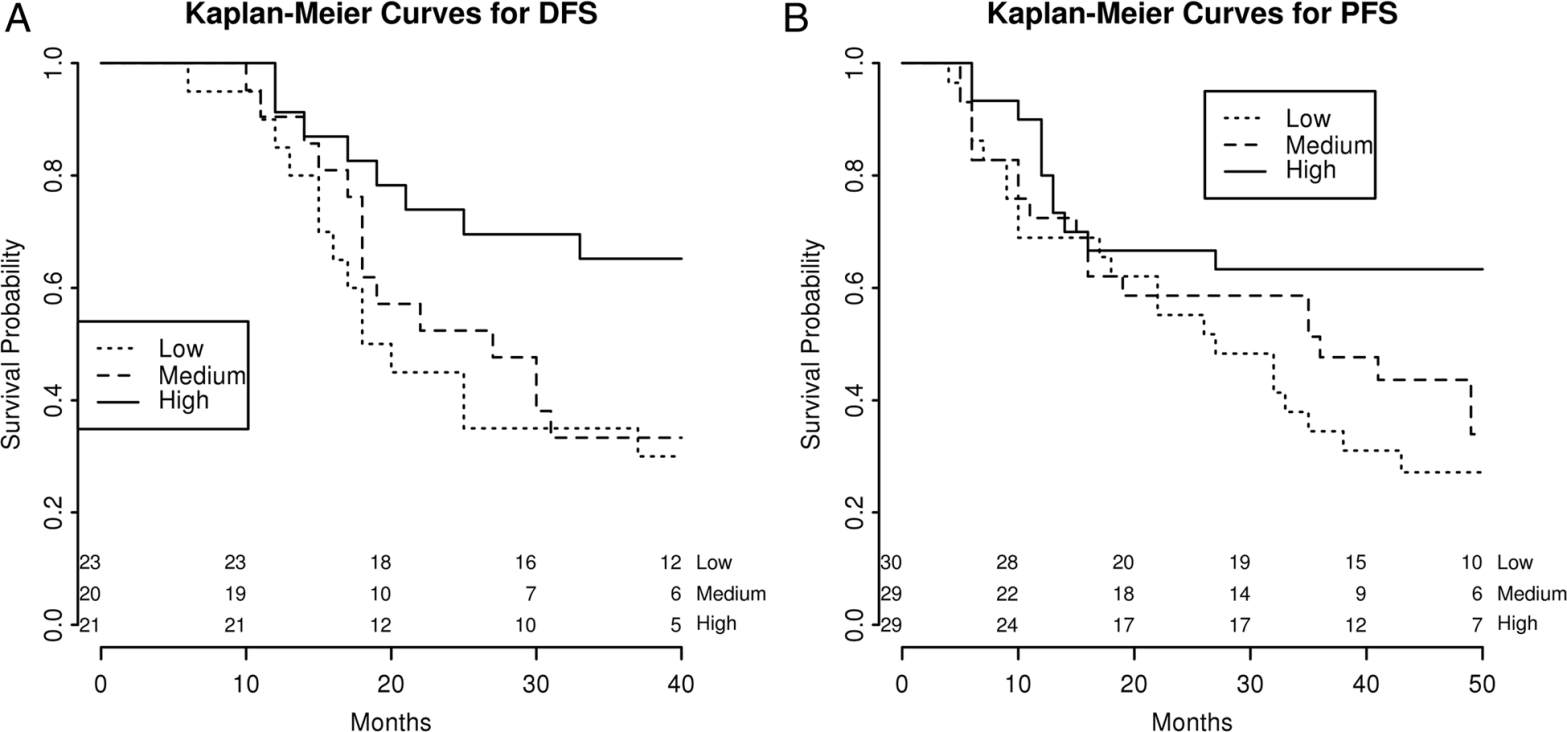
**Kaplan-Meier survival curves for EOC patients according to LipB mRNA expression. (A)** Lower LipB mRNA levels (low versus high tertiles) showed a significant association with poor DFS (p = 0.02) and **(B)** shorter PFS (p = 0.03) in the entire cohort of EOC patients.

As expected, known EOC clinical prognostic factors such as FIGO stage, residual tumor, presence of ascites and lymph node involvement showed a statistically significant association with DFS and PFS in univariate survival analyses (all p < 0.05, Table 4), demonstrative of the representative nature of the patient cohort recruited in this study. In addition, as displayed in Figure 4A and 4B respectively, lower LipB mRNA levels (low versus high tertiles) showed a significant association with poor DFS (p = 0.02) and shorter PFS (p = 0.03). FIGO stage, residual tumor, presence of ascites and lymph node involvement were of prognostic significance for disease-specific OS, whereas LipB gene expression was not. No significant correlation was found between LipB immunohistochemical staining and survival variables (Table 4).

**Table 4:** Univariate and multivariate analyses of overall survival (OS), disease-free survival (DFS) and progression-free survival (PFS) in relation to clinical parameters

***Univariate survival analysis***												
	**OS**				**DFS**				**PFS**			
**Variables**	**N**	**HR**	**95% CI**	**p**	**N**	**HR**	**95% CI**	**p**	**N**	**HR**	**95% CI**	**p**
**LipB mRNA RQ**												
Low vs high tertile	92	1.6	0.7-3.3	0.220	64	2.5	1.1-5.6	**0.020**	88	2.2	1.1-4.3	**0.030**
**LipB IHC**												
Continuous	92	1.4	0.6-3.3	0.400	67	0.99	0.3-2.4	0.980	91	1.3	0.6-2.8	0.499
**LipB IHC**												
Categoric												
IHC = 2 vs IHC ≤ 1	92	0.8	0.4-1.6	0.604	67	0.81	0.4-1.6	0.552	91	0.9	0.5-1.7	0.892
IHC = 3 vs IHC ≤ 1		1.7	0.7-3.7	0.183		1.15	0.4-2.9	0.764		1.4	0.6-3.1	0.353
**Age (years)**												
>60 vs ≤60	92	2.0	1.1-3.7	0.017	67	1.15	0.6-2.1	0.660	91	1.85	1.1-3.2	0.026
**FIGO stage**												
III-IV vs I-II	92	8.1	2.5-26.2	**<0.01**	67	6.6	2.5-17.1	**<0.01**	91	7.8	2.8-21.7	**<0.01**
**Residual tumor (cm)**												
RT > 0 vs RT = 0	92	3.7	1.8-7.8	**<0.01**	67	2.4	1.2-4.6	**0.010**	91	3.4	1.7-6.5	**<0.01**
**Presence of ascites**												
Yes vs no	92	2.6	1.4-5.1	**0.003**	67	2.8	1.4-5.4	**0.002**	91	2.6	1.4-4.7	**<0.01**
**Lymph node involvement**												
Positive vs negative	73	1.9	0.9-4.0	**0.04**	58	3.4	1.7-6.7	**0.001**	73	2.8	1.4-5.2	**0.002**
***Multivariate survival analysis***												
**Variables**	**OS**				**DFS**				**PFS**			
	**N**	**HR**	**95% CI**	**p**	**N**	**HR**	**95% CI**	**p**	**N**	**HR**	**95% CI**	**p**
**LipB mRNA RQ**												
Low vs high tertile	92	1.8	0.9-3.9	0.103	64	3.9	1.7-9.1	**0.001**	88	2.8	1.4-5.8	**0.004**
**FIGO stage**												
III-IV vs I-II	92	5.1	1.2-20.5	**0.022**	67	13.7	4.3-43.2	**<0.01**	91	5.8	1.7-19.4	**0.004**
**Residual tumor (cm)**												
RT > 0 vs RT = 0	92	1.1	0.4-2.9	0.799	67	4.9	0.07-0.57	**0.003**	91	1.02	0.4-2.3	0.964
**Presence of ascites**												
Yes vs no	92	1.6	0.8-3.4	0.180	67	3.3	1.3-8.3	**0.009**	91	1.8	0.9-3.6	0.095

FIGO stage, residual tumor, presence of ascites and LipB gene expression levels were then included in a multivariate survival analysis. As displayed in Table 4, low LipB mRNA expression and the established prognostic factors for EOC were identified as independent predictors for poor DFS (all p ≤ 0.02). Moreover, low LipB mRNA expression level, along with advanced FIGO stage, retained significance as an independent prognostic factor for shorter PFS (p = 0.004, Table 4). For OS, only FIGO stage was significantly predictive; among the other clinical parameters, LipB mRNA expression levels exhibited the highest trend toward significance (p = 0.103, Table 4).

## Discussion

Secretoglobins are small, secreted proteins increasingly recognized for their prognostic capacity in a variety of human cancers, though the precise pathophysiologic functions for most members remain to be elucidated [24]. Secretoglobins participate via one to three cysteine bonds in antiparallel heterodimerization (e.g., lipophilin B with mammaglobin A and B) [25-27]. or homodimerization (e.g., uteroglobin) with the capacity to form tetramers [10]. Such quaternary protein interactions underscore the importance of simultaneous characterization of the relative expression among multiple secretoglobin proteins. Thus, to complement previous findings by our group in EOC, suggesting that elevated mammaglobin B gene and protein expression prognosticate improved clinical outcome [23], in this report we describe for the first time relative expression patterns across a comprehensive panel of human secretoglobin family members. Secretoglobin gene expression was evaluated on 53 EOC tissues of different histologies and 30 normal controls (NO), including 10 HOSE, 10 fresh-frozen normal ovarian biopsies and 10 normal ovarian surface epithelium brushings. The choice of normal controls in ovarian carcinoma gene expression studies is still a matter of debate, since the origin and pathogenesis of this neoplasm is not completely understood.

In the present paper, we have chosen to use ovarian surface epithelial cells as controls, following the long lasting theory that, for a significant number of ovarian cancers, the pathogenesis is related to the invagination of the ovarian surface epithelium (OSE) at the time of ovulation into the underlying stroma to form inclusion cysts with a newly acquired müllerian phenotype (by metaplasia), as precursors of most of the ovarian cancer subtypes studied in our manuscript [28,29]. Consistent with this view, a recent report [30] has identified a specific transitional area of the OSE, as a previously unrecognized stem cell niche that can be susceptible to malignant transformation, providing additional implications for understanding ovarian carcinoma pathogenesis. For all these reasons we believe the controls used in our study are reasonable and in agreement with the current ovarian cancer literature.

In our analysis, compared to normal ovarian tissues, EOC specimens overexpressed mammaglobin A, lipophilin A, lipophilin B, and RYD5 genes. Based on studies of uteroglobin, the most well-characterized and founding member of the secretoglobin family, broad putative roles for these members may involve progesterone binding, immunomodulation, and activation of anti-inflammatory pathways [10]. Interestingly, HIN-1 downregulation in lung, pancreatic, and prostate cancers has been shown to correlate with cellular differentiation status [31], often via promoter hypermethylation; moreover, loss of UGB has been reported to be related to advancing grade in prostate carcinoma [32]. Those results suggest a role for certain secretoglobins as tumor suppressors, the loss of which correlates with poor prognosis [33]. Interestingly, while HIN-1 and RYD5 localize to chromosomes 19 and 11p15.5, respectively, mammaglobin A/B and lipophilin A/B map to chromosome 11q13 [5,24], chromosomal alterations of which have been implicated in poor prognosis in squamous cell carcinomas of the head / neck [34] and in the pathogenesis of some breast cancers [35].

Among EOCs, lipophilin B demonstrated not only the most marked differential gene expression compared to normal ovary, but also the strongest correlation with mammaglobin B gene expression. This relationship is not unanticipated, given that non-glycosylated LipB with a predicted molecular weight of 7.7 kDa has been shown to form covalent complexes with the heavily glycosylated 10.5 kDa mammaglobin A [18,27,36]. LipB overexpression has also been observed in malignant samples from breast, uterus, cervix, and pituitary [15-18]. Concordance of mammaglobin A and LipB mRNA expression in breast, uterine, and cervical cancers has been reported to range from 56-100% [18,36]; in contrast, no correlation has been demonstrated in kidney or prostate tissues [18]. Importantly, in the present paper we demonstrate for the first time that LipB gene overexpression has potential prognostic utility and identifies less aggressive EOC phenotypes. LipB gene expression correlated with low-grade (p = 0.02) tumors in our cohort of patients. Remarkably, lower LipB mRNA levels (low versus high tertiles) are an independent prognostic marker for decreased disease-free (p = 0.001, HR = 3.9) and progression-free survival (p = 0.004, HR = 2.8). These findings are consistent with previous reports in breast cancer, suggesting that LipB mRNA overexpression correlates with favourable factors, such as retention of estrogen-receptor positivity, low-grade disease and low proliferation rate [16]. To our knowledge, few papers have investigated the functional role of LipB overexpression in cancer cells: Sjodin *et al.* demonstrated that ectopic overexpression of lipophilin B did not affect the cell proliferation rate of breast carcinoma cells in vitro [37], while Tucker *et al.* reported that it does not appear to mediate chemoresistance to the estradiol derivative estramustine in hormone refractory prostate cancer [38]. Despite undercharacterization of function, LipB has a well-described role as a tumor-associated antigen. Serum antibodies to lipophilin B have been detected in breast and ovarian cancers at titers that reflect advanced stage and which are absent in normal controls [39]. This strong response to lipophilin B could indicate that it may exist in the sera of cancer patients in the free form, though circulating lipophilin B itself has not yet been isolated from blood.

While EOCs also overexpressed LipB at the protein level compared to normal ovary, abundance as quantified by immunohistochemistry failed to demonstrate significant prognostic associations with clinicopathological variables or survival. This result is not completely unexpected considering that, in the analysis of our cohort of patients, the correlation coefficient between LipB mRNA and protein expression was not significant. Moreover, in this regard, a discrepancy between mRNA and the encoded protein abundance in cancer has been previously reported in several studies [40-42]. This phenomenon is presumably a result of gene expression biology, suggesting various levels of regulation during protein synthesis in higher organisms (posttranscriptional regulation mechanisms involving mRNA stability, translation initiation and protein stability), likely mediated by the recently discovered class of biological molecules called non-coding RNAs.

## Conclusions

In summary, this is the first quantitative investigation of gene expression across a comprehensive panel of secretoglobins in both normal ovaries and EOC tissues. Lipophilin B, a binding partner for mammaglobin B, emerged as one of the most differentially expressed secretoglobin family members in EOC tissues relative to normal ovaries. Consistent with previous findings by this group, suggesting that mammaglobin B gene overexpression identifies less aggressive EOC phenotypes with reduced risk of recurrence, in the present study lipophilin B also functioned as an independent prognostic marker for prolonged disease-free and progression-free survival. In this way, the molecular characterization of secretoglobin expression by ovarian tumors predicts biologic behaviour and may eventually contribute to treatment algorithms. Future studies on Lipophilin B should focus upon elucidation of biologic function in order to contribute to the broader understanding of its role in EOC pathogenesis.

## Consent

Written informed consent was obtained from the patients for the publication of this report and any accompanying images.

## Abbreviations

EOC: Epithelial ovarian cancer; NO: Normal controls; HOSE: Human ovarian surface epithelial short-term cultures; FIGO: International Federation of Gynecology and Obstetrics; WHO: World Health Organization; qRT-PCR: quantitative real-time Reverse Transcription Polymerase Chain Reaction; RQ: Relative quantification; LipB: Lipophilin B; IHC: Immunohistochemistry; DFS: Disease-free survival; PFS: Progression-free survival; OS: Overall survival; HR: Hazard ratio; 95%CI: 95% confidence intervals.

## Competing interests

All authors declare that they have no competing interests.

## Authors’ contributions

ADS conceived, coordinated, designed the study, interpreted the data and revised the manuscript. EB participated in the study design, created the patients’database, reviewed medical records, interpreted the data, drafted and wrote the report. RAT helped in RNA extraction and in performing qRT-PCR experiments. SC performed statistical analyses, created the tables, interpreted the data and helped to draft the manuscript. AR supervised the research group and critically reviewed the manuscript. ER and CD performed pathological and immunohistochemical study. PT and CR helped in collecting patients’ tissue samples and data from medical records and critically reviewed the manuscript. EB and LZ helped in collecting follow-up data and critically reviewed the paper. CM and QF evaluated the clinical records and participated in the interpretation of the data. GT and ES participated in the study design, interpreted the data and critically reviewed the manuscript. SP provided funds and participated in the design of the study. DR helped in drafting the manuscript discussion and critically reviewed the paper. All of the authors read and approved the final manuscript.
